# Regulatory registration timelines of generic medicines in South Africa: Assessment of the performance of SAHPRA between 2011 and 2022

**DOI:** 10.1186/s40545-023-00537-0

**Published:** 2023-03-02

**Authors:** Lerato Moeti, Madira Litedu, Jacques Joubert

**Affiliations:** 1 South African Health Products Regulatory Authority (SAHPRA), Kirkness Street, Arcadia, Pretoria, 0007 South Africa; 2 School of Pharmacy, University of the Western Cape, Robert Sobukwe Road, Bellville, Cape Town, 7535 South Africa

**Keywords:** South African Health Products Regulatory Authority (SAHPRA), Registration, Regulatory performance, Generic products, Backlog, Finalisation, Approval time

## Abstract

**Background:**

Various regulatory authorities are experiencing backlogs of applications which result in delayed access to medicines for patients. The objective of this study is to critically assess the registration process utilised by SAHPRA between 2011 and 2022 and determine the fundamental root causes for the formation of a backlog. The study also aims to detail the remedial actions that were undertaken which resulted in the development of a new review pathway termed the risk-based assessment approach for regulatory authorities experiencing backlogs to implement.

**Methods:**

A sample of 325 applications was used to evaluate the end-to-end registration process employed for the Medicine Control Council (MCC) process between 2011 and 2017; 129 applications were used for the backlog clearance project (BCP) between 2019 and 2022; 63 and 156 applications were used for the risk-based assessment (RBA) pilot studies in 2021 and 2022, respectively. The three processes are compared, and the timelines are discussed in detail.

**Results:**

The longest median value of 2092 calendar days was obtained for the approval times between 2011 and 2017 using the MCC process. Continuous process optimisation and refinement are crucial to prevent recurring backlogs and hence implementation of the RBA process. Implementation of the RBA process resulted in a shorter median approval time of 511 calendar days. The finalisation timeline by the Pharmaceutical and Analytical (P&A) pre-registration Unit, which conducts the majority of the evaluations, is used as a tool for the direct comparison of the processes. The finalisation timeline for the MCC process was a median value of 1470 calendar days, the BCP was 501 calendar days and the RBA process phases 1 and 2 were 68 and 73 calendar days, respectively. The median values of the various stages of the end-to-end registration processes are also analysed in order to build efficiency within the process.

**Conclusions:**

The observations from the study have identified the RBA process which can be implemented to reduce regulatory assessment times while assuring the timeous approval of safe and effective, quality medicines. The continuous monitoring of a process remains one of the critical tools required to ensure the effectiveness of a registration process. The RBA process also becomes a better alternative for generic applications that do not qualify to undergo the reliance approach due to its drawbacks. This robust procedure can therefore be utilised by other regulatory agencies that may have a backlog or want to optimise their registration process.

**Supplementary Information:**

The online version contains supplementary material available at 10.1186/s40545-023-00537-0.

## Background

In the effort to reduce the likelihood of a backlog of medicinal product applications, which has the propensity to build up in medicine regulatory bodies globally, the performance of regulatory review should be measured and tracked [1]. The need for agencies to measure and improve their performance proactively and consistently against stated target times is one of the World Health Organization (WHO) global benchmarking tool parameters [2]. This is especially important for generic products as they increase accessibility and affordability in global healthcare systems. Generic products contain the same quantity of active substances in the same dosage form, meet the same or comparable standards and are intended to be administered by the same route as the innovator products [3]. In most countries, these generic products are marketed only after patent expiration and are normally cheaper than branded innovator medicines [4].

In 2015, China’s Food and Drug Administration (CFDA) had more than 21 000 applications in backlog, most of which were generic products [5]. In 2019, the CFDA's 900-day approval period was shortened to 300 days [5]. Their Centre of Evaluation (CDE) employees expanded from 100 in 2015 to approximately 1000 by 2020; this was reported as one of the direct causes of the decline [6]. The increase in human resources, amendments to the 2007 administrative measures and processes for Drug Registration as well as the introduction of additional review pathways were implemented which accelerated access to medicines [6]. The regulatory authority in Brazil, Agência Nacional de Vigilância Sanitária’s (ANVISA) also reported that in 2018 there were more than 800 New Chemical Entities (NCE) and generic applications in the backlog with the intent to clear the number by January 2019 with improved registration processes [7]. ANVISA had achieved an approval time of 795 days for generic products in 2013–2016 for 138 products. [1] The United States Federal Drug Administration (USFDA) on the other hand accomplished an approval time of 661 days in 2020 for 737 Abbreviated New Drug Applications (ANDA) approvals and 172 ANDA tentative approvals [8], while the Australian regulatory authority, Therapeutic Goods administration (TGA) accomplished an approval time of 244 calendar days for 85 generic products in 2021 [9]. This shows that the approval times are dependent on the number of applications received in that specific year and the resources available in the authority. The Taiwan Food and Drug Administration stated that they receive an estimated 400 generic applications per annum [10]. The Caribbean Regulatory authority received 11 generic applications in 2018 [11], TGA received 85 applications in 2021 [9] and South African Health products regulatory authority (SAHPRA) received an annual average of 1247 applications in 2019 [12]. It is therefore the duty of the authorities to ensure that the required measures, review tools and developed processes that best suit the situation they are faced with are continuously monitored and efficiencies applied.

The South African authority, SAHPRA, formerly named the Medicine Control Council (MCC) reported a backlog of approximately 8000 applications in 2016 which highlights the need to review the registration process and apply better efficiencies [13]. The authority had a fast-track process initiated in 2003 which only focused on essential and critical medicines [14]. Due to the backlog that formed, a number of medicines in the essential list were fast-tracked, therefore only these products were allocated and evaluated while other products were allocated only when an evaluator was available. Given that the human resource was at a minimal and a registration process had not been reviewed for more than 20 years, the backlog increased [14]. The operational challenges and resource constraints faced by SAHPRA over the years resulted in the formation of a backlog of approximately 16 000 applications including variations by 2018 [15]. In 2019 when the backlog clearance project (BCP) was initiated, 15 domestic and 48 international evaluators were contracted to assess the quality and bioequivalence assessments while SAHPRA’s business-as-usual section operates as normal with the new applications received [16]. This strategy would allow for the authority to function while the backlog is managed as a separate project with the required human resource employed to execute the required end-to-end backlog function. This was aided through the assistance of funding from various entities such as the Bill and Melinda Gate Foundation and the National Treasury of South Africa. This meant that careful monitoring and consistent reporting was required to ensure that the project’s goal was executed. With funding acquired and after an in-depth analysis of SAHPRAs backlog by a project managing consulting firm, a target completion time of two years was predicted based on the available resources [16]. This was not executed as planned and it was extended by one year and 4 months [17].

This study, therefore, investigates the end-to-end registration process of generic products employed between 2011 and 2022 for the MCC process and the BCP process in the effort to assess the performance and identify the root causes of the backlog. In addition, the developed robust pathway called the risk-based assessment (RBA) process with remedial steps implemented to mitigate future backlogs is described and compared with the other processes.

## Methods

The study assesses three different registration processes used between 2011 and 2022; the MCC process is assessed using a sample of finalised applications between 2011 and 2017; the BCP process is assessed using the applications from three re-submission windows (RW) evaluated in 2020; and the RBA pilot studies assessed in 2021 and 2022 using the sample of applications that were in RW8, 10, 11 and 12. The RBA approach is the robust process that was developed upon further refinement and optimisation of the MCC and BCP process and piloted in 2021 and 2022, titled the RBA pilot study phase 1 and 2.

### MCC registration process, 2011–2017

Over the 7-year period, 3148 applications were finalised by the P&A pre-registration Unit within SAHPRA of which 2089 were non-sterile. Thus, due to the large application size at hand, a statistical sampling method became a requirement for this research. The sample selected becomes a true representation of the population and results of the study can be generalised to the population. The method of selection and calculation of the representative sample is comprehensively described by Moeti et al. where a sample size of 325 non-sterile products is obtained and used in the study [13, 18, 19]. By comparing the quality requirements for sterile and non-sterile products it is witnessed that the sterile products require additional assessments in the pharmaceutical development Sect. (3.2.P.2) as well as the process validation and or evaluation Sections (3.2.P.3.5). On the other hand, the non-sterile products would normally require additional assessment in the regional section on bioavailability, therefore, assessment times would be similar for both product types.

### Backlog clearance project (BCP) registration process, 2019–2022

In order to eliminate the backlog, in 2019 SAHPRA started a project named the BCP [19]. The project was initiated with ~ 8220 applications in the pre-registration phase [16]. The implemented process allowed for applicants to re-submit the dossiers, as some information may be outdated since they were submitted as back as 2008. Resubmission windows (RW) were then created according to therapeutic categories with those considered essential in the earlier windows.

The applications selected from the BCP were from three RWs, i.e. RW1, RW5 and RW6. RW1 consisted of medicines in the therapeutic category of Human Immunodeficiency Virus (HIV), tuberculosis (TB), vaccines and hepatitis, RW5 was for medicines targeting diabetes, malaria, maternal and new-born health as well as all the priority APIs and RW6 was for medicines targeting respiratory system diseases [20]. An overall of 129 applications from the three windows was employed and only the applications that utilised the full review pathway for quality and bioequivalence scientific assessments were selected. Note that other pathways include the reliance pathway [21] or applications that have previously received preliminary approval from the P&A pre-registration Unit, however, not yet registered and contained minor variations. Since the approval times for these pathways were shorter, this would alter the calculated timeframes, therefore, the applications that undertook the reliance route were not included in the study. The dates at each stage of the BCP registration process for each application were collected from the electronic database/tracker used by the authority.

### Risk-based assessment (RBA) pilot study, phase 1 and 2, 2021–2022

The risk-based pilot project was initiated in September 2021 within the realm of the BCP using 63 applications from (RW8) as they were next in line to be allocated for initial full review. RW8 comprised of medicines in the therapeutic category that treats haematological/immunological diseases as well as medicines that are analgesics and Non-Steroidal Anti-Inflammatory Drugs (NSAIDs). For further optimisation and reproducibility of the process, the RBA pilot study was up-scaled in April 2022 using 159 applications from RW 10, 11 and 12. The therapeutic categories are; endocrine, nutritional, digestive system and metabolic disease for RW10; skin, subcutaneous tissue, musculoskeletal system and connective tissue for RW11; and eye and ear diseases for RW12 [20]. The implementation was made as an intervention to promote efficiencies within the existing registration process and allow accelerated access to medicines. The dates were collected from the database created during the initiation of the pilot studies wherein all activities and dates were recorded and closely monitored at each stage.

The dates were collected and information was populated in the respective Microsoft Excel®, 365, Worksheets. The differences between each activity were calculated for each product and median values were calculated for each, to obtain the time it takes for each activity within the registration process. Finalisation is the conclusion of an assessment by each respective Unit before registration. It should be noted that the finalisation timeline by the Pharmaceutical and Analytical (P&A) pre-registration Unit, is used as a tool for the direct comparison of the processes as the Unit is assessing the bulk of the information submitted by the applicant.

## Results

### Brief description of the MCC, BCP and RBA processes

**Fig. 1:**
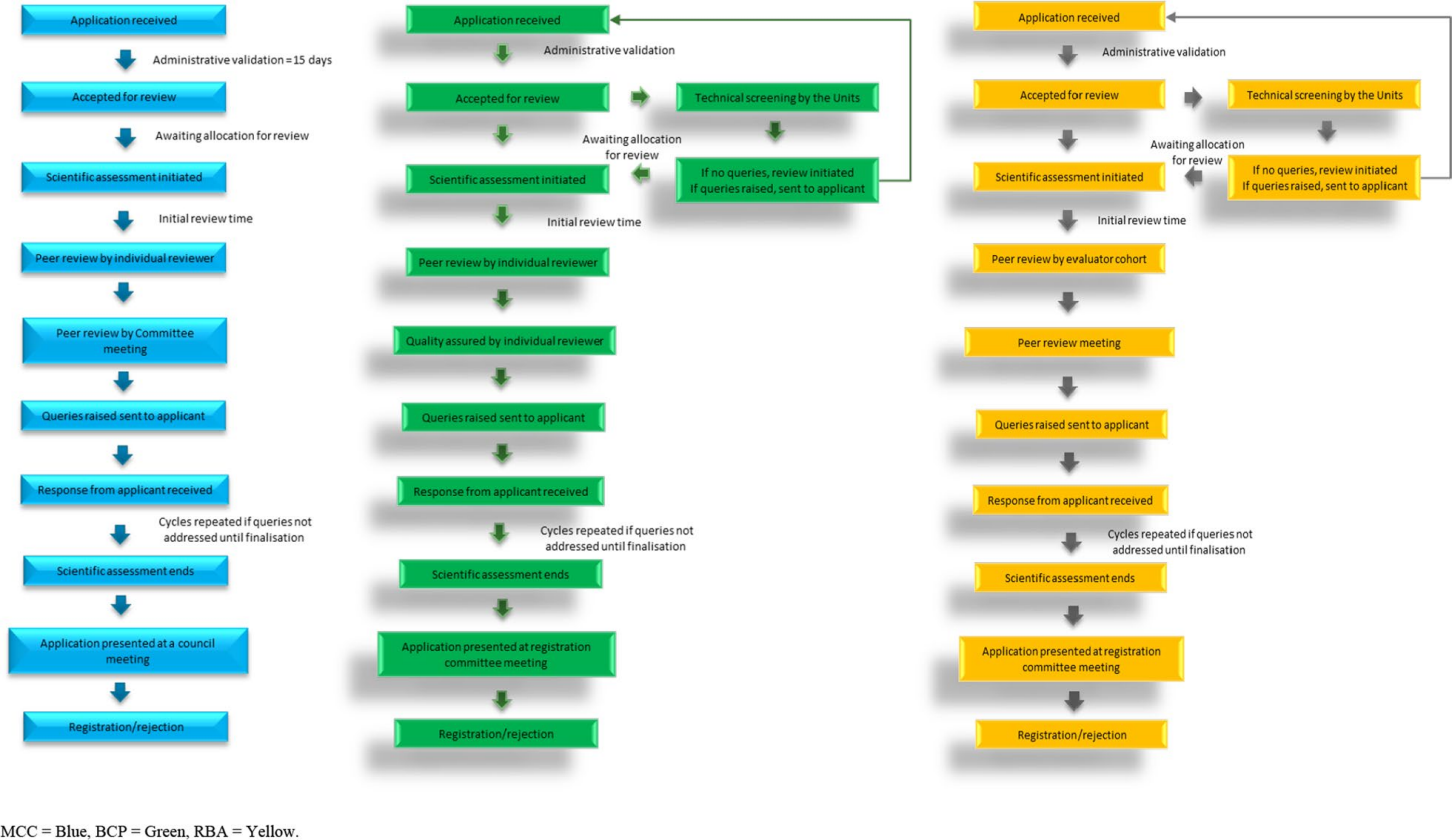
Depiction of the MCC, BCP and RBA processes utilised by SAHPRA between 2011 – 2022. MCC = blue, BCP = green, RBA = yellow

The registration processes remain largely similar with deviations observed in certain steps as highlighted in Fig. 1. Upon receipt of the application, administrative screening was performed within 15 calendar days from the time of receipt for the MCC process. Applications were then routed to the relevant Units, where they are allocated to an evaluator to start the review process for the MCC process while for the other two processes technical screening was performed as illustrated in Fig. 1. Queries raised from the technical screening were sent to the applicant and a response was requested within 10 working days. When all queries were addressed or the application is compliant the allocations for scientific assessments were initiated based on evaluator availability. Due to the limited number of evaluators, the application would wait in queue for an available evaluator before allocation. Once allocated in the P&A pre-registration Unit, the initial scientific assessments were conducted. The peer review stage differed in the three processes as shown in Fig. 1 in that detailed assessment reports prepared by the evaluators were peer-reviewed by the Chair or deputy Chair of the Committee in the MCC process. Thereafter, these were made part of the agenda and shared with the Scientific Committee members for discussion during the meetings held every 6 weeks. In the BCP process, reports were peer-reviewed by an individual peer reviewer and thereafter quality assured by another assigned evaluator based on individual evaluator availability. In the RBA process, once the detailed assessment reports were received from the evaluators, the When Available poll [23] was used to determine the most suitable time for each weekly peer review session. The reports were compiled into meeting documents and uploaded on Google Docs [24] well in advance (5–7 days) to allow evaluators to provide their comments during peer review [22]. The peer review meeting sessions were then held and only specific points of discussion, highlighted by the peer review panel, were discussed.

In the P&A pre-registration Unit, recommendations pertaining to quality and bioequivalence data were sent to the applicant and a response was expected within 90 calendar days for MCC process, 20 working days for BCP process and 15 working days for initial queries and 10 working days for response queries for the RBA process. The response would be reviewed by an evaluator and undertake the peer review process as described for each process. There were no limits to the number of response cycles between the applicant and the authority in the MCC process while this was restricted to only 2 response cycles for the BCP and RBA processes. Once the application is finalised by the P&A pre-registration Committee, the Clinical Committee, Good Manufacturing Practices (GMP) Committee and the Names and Scheduling Committee or their Units thereof, the medicine is considered for registration/approval by the authority at a Council meeting held every 60 calendar days in the MCC process or registration Committee meeting held weekly for the BCP and RBA processes.

### Reported timelines for the three processes

**Fig. 2:**
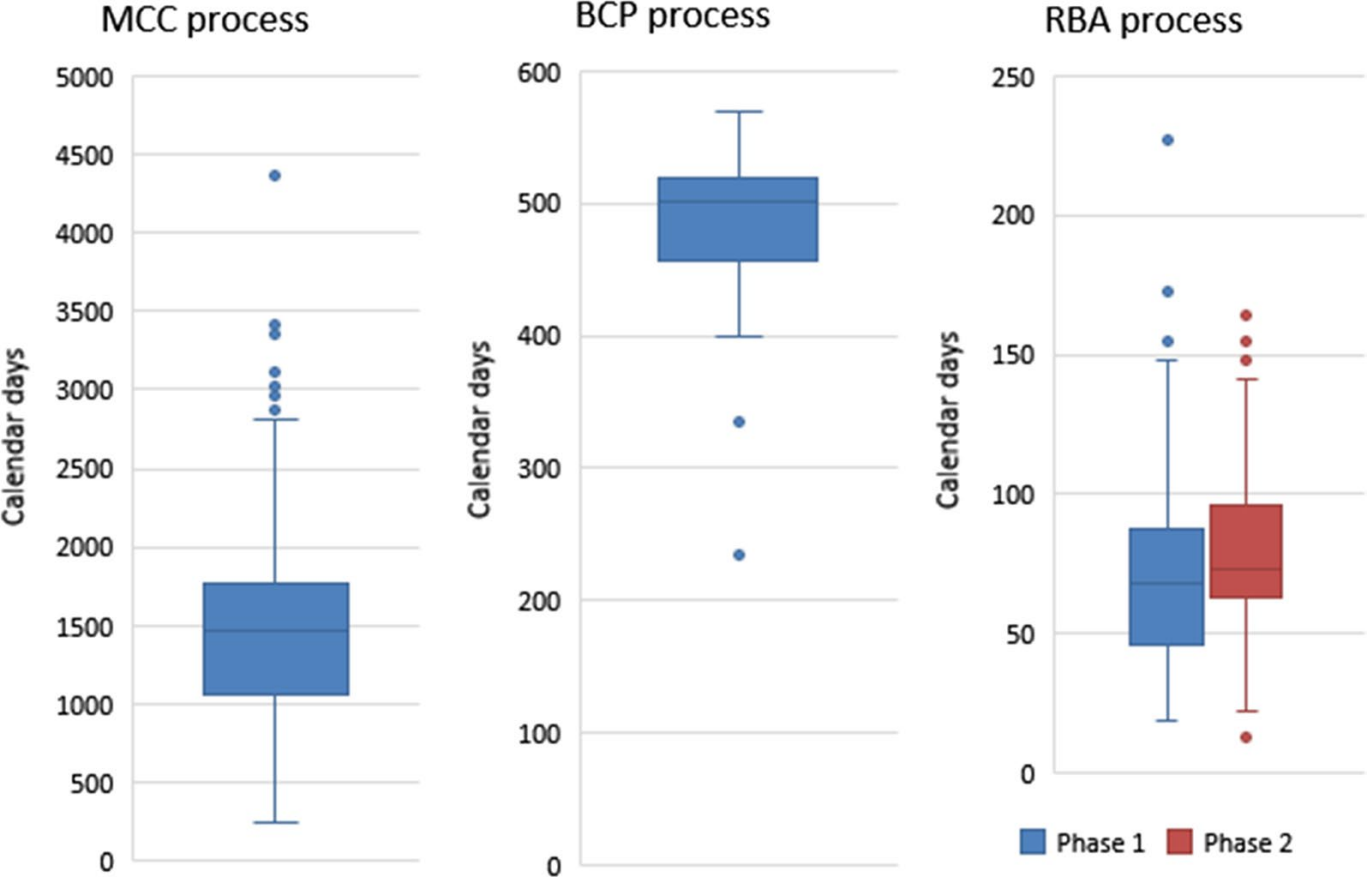
The graphical representation of finalisation timelines for the MCC, BCP and RBA processes with reported median values of 1470, 501 and 68 calendar days, respectively. *n*; MCC = 325, BCP = 129, RBA Phase 1 = 59 (4 applications were withdrawn before finalisation), RBA Phase 2 = 156 (3 applications were withdrawn before finalisation)

The median values at each stage in the P&A pre-registration process were calculated and are depicted in Table 1 for all the different end-to-end registration processes. Figure 2 illustrates the overall median finalisation time for the MCC, BCP and RBA processes as 1470, 501 and 68 calendar days. The second phase of the RBA pilot study was conducted in 2022 and the reported median finalisation time was 73 calendar days which is relatively similar to Phase 1. The results for RBA pilot study phase 1 and 2 as depicted in Table 1 confirm similarity for each timeframe.

**Table 1:** The identified activities within the three end-to-end registration processes employed by SAHPRA between 2011 and 2022 and the median timelines of the activities

	Allocation timeframe	Preparation of assessment reports	Peer review process	Quality assurance	List of queries to the applicant	Applicant time
Cycle	Median time in calendar days for registration activities for the MCC process (2011–2017)					
1	682	201	–	-	74 (0 finalised)	347
2	186		62	–	72 (168 finalised)	76
3	56		76	–	74 (116 finalised)	76
4	31		47	–	32 (35 finalised)	56
5	16		16	–	20 (6 finalised)	-
	Median finalisation timeline			1470		
	Median registration timeline			2092		
Cycle	Median time in calendar days for registration activities for the BCP (2019–2022)					
1	278	63	29	35	30 (0 finalised)	84
2	22	35	15	30	15 (30 finalised)	33
3	10	30	10	20	15 (58 finalised)	22
4	7	7	5	10	10 (25 finalised)	20
5	2	11	5	15	5 (13 finalised)	–
	Median finalisation timeline			501		
	Median registration timeline			591		
Cycle	Median time in calendar days for registration activities for the RBA phase 1 pilot study (2021–2022)					
1	431	5	8	–	2 (3 finalised & 2 withdrawn)	25
2	2	2	6	–	1 (44 finalised)	18
3	1	1	7	–	1 (6 finalised & 2 withdrawn)	10
4	1	1	7	–	1 (4 finalised)	–
	Median finalisation timeline			68		
	Median registration timeline			511		
Cycle	Median time in calendar days for registration activities for the RBA phase 2 pilot study (2022)					
1	~ 2 years	5	8	–	1 (6 finalised)	28
2	2	2	7	–	1 (102 finalised & 1 withdrawn)	15
3	1	1	7	–	1 (44 finalised & 2 withdrawn)	12
4	1	1	5	–	1 (7 finalised)	–
	Median finalisation timeline			73		
	Median registration timeline			–

In the MCC process, the first row of Table 1 represents cycle 1, where column 2 reflects the median time for the number of calendar days from the date the application was received to the time it was allocated for assessment as 682 calendar days. The time taken from the allocation of the application to the evaluator to the time the initial report was submitted is 201 days as indicated in column 3, the time taken from when the report was submitted to the initial peer-review meeting is 171 days, and from peer-review meeting to the time the query letter was sent to the applicant is 74 days. The last column indicates that it took the applicants 347 calendar days to respond to the initial queries, despite being granted only 90 calendar days to respond. This demonstrates how the applicants were also responsible for the delays. It emerged that some applicants would ask for extensions to provide the necessary data, which were granted, while others would exceed the response limit without asking for an extension. Due to the difficulty in obtaining the allocation dates of the responses for cycles 2 through 5 as depicted in Table 1, the time when responses were received to when report was submitted are merged. This is because the dates on which the responses were allocated to the evaluators were not recorded. The MCC process took up to five cycles before a product was finalised for the selected representative sample.

To assess the BCP process, the first row under the BCP median times in Table 1 represents cycle 1, which reflects the median time from the date of receipt to allocation for assessment as 278 calendar days. The time taken from allocation to submission of initial report is 63 days, the time taken from submission of report to the initial peer-review is 29 days, and from peer-review to quality assurance (QA), is another 35 days. The time taken from QA to sending the query letter is 30 days with the applicant taking 84 days to respond to the queries.

For the RBA process, the first row under the RBA median times in Table 1 represents cycle 1, which reflects the median time from the date of receipt to allocation for assessment as 431 calendar days while phase 2 denotes 523 days. The time taken from allocation to submission of initial report is 5 days, the time taken from submission of report to the initial peer-review meeting is 8 days for both studies, and lastly, from peer-review meeting to communicating the query letter to the applicant is 1–2 days. Table 1 also outlines the number of applications finalised or withdrawn in each cycle in column 6. For example, in cycle 1 of the RBA process phase 1, three (3) applications were finalised and two (2) were withdrawn while 6 were finalised in RBA phase 2. Cycles were repeated four times depending on the queries and whether the response from the applicant was compliant or not.

## Discussion

### Alternative regulatory review models

Authorities use different regulatory review models to expedite access to medicines. These review models include the use of reliance strategy, whereby a regulatory authority in one country may consider and give significant weight to scientific assessments or inspection reports performed by another authority or trusted institution. Verification, abridged, and mutual recognition models are the reliance approaches that are used. Abridged review model is a selective assessment of market authorisation data, provided the product is registered by a reference national regulatory authority (NRA) [25]. This sort of study focuses on country-specific product quality requirements and clinical data for benefit–risk analysis. Verification model allows NRAs to rely on another NRA's regulatory decision by only comparing the submitted data which speeds up regulatory review [25]. SAHPRA implemented reliance models in 2019 and it was anticipated that using the verification and abridged review methods for most generic applications would reduce the backlog, however, this was not the case. SAHPRA considers the following countries as reference NRAs: USFDA, the European Medicines Agency (EMA), individual EU member states, the Japanese Pharmaceuticals and Medical Devices Agency (PMDA), Health Canada, Swissmedic, the Medicines and Healthcare products Regulatory Agency (MHRA) of the United Kingdom, and the Australian Therapeutic Goods Administration (TGA) [16]. From this pool of authorities, full unredacted assessment reports are required to confirm the review. The limitations of this model include:

- In some circumstances primarily when European countries are NRAs, applicants have the reports and would share these with the authority, however, in most cases, these would not be available. The applicant must subsequently submit a letter confirming similarity to the reference country's application. The process of obtaining the reports from the NRAs takes months as they have other priorities. In other cases, the NRA requires the principal marketing authorisation holder to submit a declaration of access for the applicant in South Africa for authorisation of sharing the reports which would often take months before receipt of the reports. These result in delays in the registration timeframes
- From the reports shared, it is evident that the majority of the submissions had undergone numerous variations without amendments approval letters. The applications will then be subjected to full review and this will constitute more work as the information from the other regulatory authority required validation.
- Generic, well-known, pharmacopoeial applications registered in NRAs without unredacted reports will undergo a full review. Due to the absence of the reports from the NRA, a comprehensive review would be done even though these applications pose a negligible risk based on the aforementioned characteristics. This approach wastes scarce resources for an organisation with significant resource constraints, necessitating the need for an alternative strategy for such applications.

As a result of the abovementioned drawbacks, approximately 20% of the applications were legible for the reliance pathway and the rest had to be subjected to full review. The RBA model is intended to deal with well-known generic applications that do not qualify for reliance review [22]. In-depth discussions are made for each stage of the registration process to identify obstacles and root causes of the backlog and how they were addressed by the RBA strategy to expedite the registration process.

### Allocation timeframe

The median value from receipt of dossiers to allocation for assessment is 682 calendar days for the MCC products finalised between 2011 and 2017, this is considerably higher compared to ANVISA with 214 calendar days for applications approved between 2013 and 2016 [1]. Insufficient human resources resulted in time-lapse of approximately two years from receipt to allocation of the dossiers. Regular applications received in 2012 were only being allocated in 2016 [13]. This demonstrates that since the fast-tracked applications received priority for evaluation, the waiting period for the regular applications was 4 years in 2016 [14]. These delays had resulted in a backlog of 7902 applications in 2016. To eliminate the backlog, in 2019 SAHPRA started a project named the BCP as described in the section above [19]. Due to this, the date of receipt for applications in the BCP and RBA pilot study are the re-submitted dates. These are reported as 278 and 431 days, respectively. The difference in these times is attributed to the different times that were allocated for the various re-submission windows. For instance, RW1 was resubmitted between 01 August 2019–30 September 2019 while RW8 was resubmitted between 01 July 2020 to 30 July 2020 which is almost a year later [20] (see Additional file 1). Applications in earlier windows were assessed first while applications in later windows awaited the availability of evaluators. Although the median values of 278 and 431 days are quicker compared to that of the MCC timeline of 682 days, they remained to be higher than those of ANVISA with a timeline of 214 days. Apart from ANVISA there has been no other reports on timelines for each stage of the registration process by regulatory authorities. Even with the improved process and re-submissions SAHPRA implemented with the BCP, it was unable to reduce this timeline to a minimum which is what the authority would need to work on improving for reduced turnaround registration times.

### Preparation of assessment reports

The time difference between the date the application was allocated for review to date when the report was received essentially determines the time it took to conduct the scientific assessments. Once the products were allocated for scientific review, the MCC process took approximately 201 calendar days to evaluate the quality and bioequivalence aspects of the dossier. A number of factors resulted in this time difference. These are highlighted below:

- The sample selected is on non-sterile products which require the evaluation of both the quality and bioequivalence studies. The available evaluators either had expertise in either one of the areas or both, therefore allocation of these would in most cases be to two different evaluators. Due to the different rates and initiation times of evaluation, one evaluator would have completed a quality assessment while another would not have started the bioequivalence assessment, or vice versa, since the allocations were conducted in bulk and were not monitored.
- The authority had a lack of skilled staff to conduct the scientific reviews and largely used external evaluators. The PEM, P&A pre-registration Unit utilised 15–20 quality evaluators and only 8–10 bioequivalence evaluators. This also led to having more quality sections evaluated while the bioequivalence sections were outstanding in some cases, thus delaying the evaluation times further.
- Once applications were given to the evaluators, there was little to no supervision of them; thus, an evaluator would work on an application for a long time without authority oversight. This led to the inability to track applications during the review process due to the lack of an efficient document management system.
- Since the external evaluators had primary work, they could only evaluate limited number of applications in their free time.

The time gap from first allocation to the time the report was received was substantially reduced from 201 to 63 calendar days for the BCP timeframes due to careful monitoring to achieve the project's aim of clearing the backlog in two years. This demonstrates how important it is to carefully oversee the registration process from beginning to end, especially in the P&A pre-registration Unit. This was also facilitated by the fact that there were more than thrice as many evaluators (63) employed to carry out the assessments as there were for the MCC process. The BCP also changed the assessment tools used which impacted on the review times. The timeline was further reduced to five (5) calendar days in the RBA phase 1 process utilising only 10 evaluators for the 63 applications and 17 evaluators for 159 applications in RBA phase 2. The five days were sufficient for the evaluators to submit their assessments owing to the strategic bulk allocation process that was used with identified similarities of applications. On average, 2 to 3 applications each week were allocated, and the evaluators would submit all the reports at once. RBA employed meticulous and thorough monitoring of each stage of the process as well as strategies to refine and reduce the review timelines. The implementation of the risk-based approach by SAHPRA is extensively reported on by Moeti and colleagues [22]. The report includes the evaluation timelines which are lower compared to the two processes detailed above.

A trend is observed with response cycles with the timelines becoming shorter as the cycles increase. For cycles 2 through 5, the MCC process had median values of 186, 56, 31 and 16 days from the time the response was received to the completion of the evaluation report, whereas cycles 2–4 for the RBA process saw a reduction with median values of 4, 2 and 2 days. The median evaluation time for the responses was also reduced to about three hours for initial responses. The RBA process evaluated the responses internally to effectively shorten the timelines compared to when external evaluators are assigned. The use of internal staff was, therefore, cost-saving.

### Peer review process

The MCC process involved an additional individual peer review to be completed prior to the committee's peer review meeting, which contributed to 171 calendar days to the time taken to peer review the initial reports that were received. EMA reported on their target assessment time of up to 120 working days for initial reports which incorporates the review and peer review process while ANVISA reported 19 days for assessment and peer review [1, 26]. The combined timelines are much shorter compared to that of the MCC process. The reports from the MCC process were peer-reviewed after the evaluations were concluded by the Chair or deputy Chair of the Committee before being discussed at the Committee meeting. This meant that the peer reviewer would need to get the hard copy dossiers to conduct an in-depth review of all the applications. Upon completion, the meeting documents were compiled and couriered to the Committee members, who also reviewed the documents independently. The P&A Committee met every six weeks, which limited the number of meetings to six or seven per year, each lasting 3.5 days, and during which the product conclusions were made. As a result, there were delays as limited reports could be discussed for one peer-review meeting session.

Since the MCC process produced a median value of 171 calendar days which is over six months, it was necessary to modify it and employ a monitoring mechanism in order to shorten this timeline. The BCP process, therefore, amended the peer review process and included a one-person peer review as well as a one-person quality assurance approach. The Committee meeting setup which promoted collaborative scientific decision-making was removed from the process. The median timeline was reported as 29 days from the period when the report was received to when it was allocated for peer review; 35 days from the period when the report was peer-reviewed to when it was assigned for quality assurance; and 30 days from the period when quality assurance was initiated and concluded. This is an overall median time of 94 calendar days for the peer review process employed in the BCP process. The refined BCP process suffers some drawbacks such as lengthy non-standardised queries to the applicant which resulted in requests of multiple extensions to respond to queries raised by the authority. In addition, significant inconsistencies in the queries were observed; applicants would receive different queries for similar products as different reviewers were used and inappropriate peer review was conducted. This also led to significant delays in registration times.

The peer review meeting approach, which is also employed by the USFDA and EMA was reinstituted in the RBA process [26, 27]. Weekly peer reviews were held, allowing for a quicker flow of query letters to the applicants. The peer review meetings provided evaluator alignment in terms of the review criteria used. These sessions also played an important role in facilitating thorough scientific debate regarding the queries raised by the primary reviewer, based on the risk to the product in question. The approach required the peer reviewers to apply analytical thinking and research skills to determine the relevance of the initial queries based on the data provided and type of application, as well as its risk to the end user. Soliciting multiple experienced reviewers to provide peer reviewer input was effective, as it ensured thorough review of all critical quality attributes, which, in turn, offered assurance that only products of high quality, safety and efficacy were approved. The timeline was significantly decreased to 10 calendar days in the RBA process. Given the expertise of evaluators employed, the meetings acted as a platform for peer review and quality assurance. The When Available poll [23] was used to determine the most suitable time for each peer review session based on the evaluators' availability. The reports were then compiled into meeting documents and uploaded on Google Docs [24] well in advance (5–7 days) to allow evaluators to provide their comments [22]. The living document would then show all comments in real-time, allowing all evaluators to see each other’s comments and refer to the electronic version of the dossier on the regulatory agency reviewing software, EURSNext, when required. This assisted in drastically reducing the meeting sessions as only specific points of discussion, highlighted by the peer review panel, were discussed. Most other aspects were collaboratively deliberated on during the real-time discussions via the Google Docs. This approach further minimises the risk as multiple assessors peer-review an application and can comment on the notes made by other peer reviewers which further facilitated review and reduces registration time considerably.

### List of queries to the applicant

In the MCC process, a median value of 74 calendar days, which is significantly high, was observed between the time when the peer review is completed to when the query letter is issued. Without detailing the peer review process, ANVISA claimed a time difference of 19 calendar days for this stage [1]. Once the peer review meetings were concluded in the MCC process, query letters were created using the meeting minutes. Lack of oversight and control resulted in the P&A Unit exceeding the targeted 14 calendar days for this step.

Since the peer review meeting approach was not used for the BCP, this timeline is not provided; nonetheless, the determined median value from the date of receipt of the quality assured report communicating the deficiencies observed was 30 calendar days, whereas the median timeline for the RBA process was two (2) days for this timeframe. This step required proper planning and preparation. The internal evaluators who coordinated the peer review meetings ensured that the query letters were prepared well in advance and amended as reviewers made comments in the live Google Docs. After the meeting, the letters are revised based on contentious issues, which takes a few hours before being forwarded to the Portfolio coordinator (PC). The applicant would then receive the query letters from the PC. A delay of one day is observed which can be improved to ensure that the PC shares the query letters immediately upon receipt.

### Applicant time

The analysis revealed that the calculated median value was 347 calendar days instead of the 90 days that was requested for response to the query letters in the MCC process. Given that ANVISA claimed a median response time of 120 days [1], this is noticeably excessive. EMA also allocates a response time of 3–6 months to the applicant once the clock-stop is paused [26]. There were numerous extension requests and a lack of response monitoring tool to easily identify when the target time is exceeded. Therefore, in some instances, the applicant would surpass the time without requesting extensions which led to a significantly high median value. This demonstrates the criticality of an effective monitoring tool at each stage of the process. The PCs were, therefore, introduced in the BCP and RBA process, to monitor and identify when the target time is exceeded.

The response timeframe was shortened to the 20 working day target period in the BCP from the 90-day target of the MCC process, however, the median timeline of 84 calendar days was obtained. For the RBA process phase 1, the calculated median value for the initial response from the applicant was 25 calendar days, with a target response time of 15 working days. The difference in RBA response times for cycle 1 (25 days), cycle 2 (18 days), and cycle 3 (10 days) and a similar trend for phase 2 was attributed to the initial queries receiving a 15-working-day response window taking in cognisance, the magnitude of the queries raised, while subsequent queries received a 10-day response window. The applicant's response time largely depended on the type of queries recommended; if significant adjustments are suggested, they requested a longer extension which was granted, and this resulted in a longer approval time.

### Response cycles and delaying queries

If the queries raised in the query letters are not addressed, the response cycles would repeat. The authority did not set a limit on the number of response rounds in the MCC process, which slowed down the finalisation timeframe. The average response cycles were five, and the maximum period for an application to be approved was 4361 calendar days. Lack of monitoring and control allowed some applications to go unattended until the applicant inquired about the status of the application.

The other aspect which led to multiple response cycles is common deficiencies observed in the quality and bioequivalence study evaluations which resulted in back-and-forth communication with the applicant [13, 18, 19]. The deficiencies in the specification sections of the API and FPP were the most prevalent and included requests to tighten the proposed specifications of the product. In such cases, the applicant would provide a justification for retaining the proposed specification, but the authority would either decline or request additional supporting data, resulting in extended cycles. These were particularly common for tightening impurity limits, assay limits, and dissolution limits, when applicable. The applicant would offer the justification listed below for not tightening the proposed specifications:

- Request to gain further experience of the product and obtain data from future batches to be manufactured before tightening the specifications.
- Justifying retaining the limits based on the results observed in the stability data.
- Justifying retaining the assay limits based on the limits stated in the pharmacopoeia when the submitted results show that the percentage label claim of not less than 95.0% can be attained for the lower limit.
- Justification to use specifications that are wider than the bioequivalence batch results.

These were some of the justifications provided that were not accepted by the authority. The specifications are set and proposed based on the submitted data, any specifications wider would not be accepted since batch-to-batch consistency and reproducibility should be maintained throughout all future batches manufactured compared to the initial validation and bioequivalence batches.

The stability sections also had recurring deficiencies such as the request for further stability data to support the proposed retest or shelf life. These fell under the common deficiencies reported by SAHPRA and are discussed extensively in the recent publications [13, 18, 19]. The response cycles would be shortened as all requirements could be met with the approach of informing manufacturers of the common deficiencies identified.

### Final adoption for registration

Once the product was finalised in the MCC process, it was sent to the administrative Unit to be collated with outcomes from the other Units before it can be registered. The median value for this stage was calculated as 482 days. This was attributed to the following:

- The initiation of evaluations was conducted at different times therefore finalisation within Units was not synchronised.
- Finalised product history packs were not sent to the administrative Units immediately upon finalisation.
- The inspections were undertaken after the P&A pre-registrations and Clinical evaluations Units completed their scientific assessments. Historically, the assessment process has been lengthy, and sites may not be GMP-compliant at the time of approval; hence, inspectors opted to perform inspections after assessments were complete. If the result was a negative GMP status, an inspection had to be rescheduled, which slowed registration, and in certain cases resulted in a rejection if the manufacturer did not meet the required GMP standards.

The following serve as potential solutions to obtain a reduced median registration time for this step:

- Sending queries simultaneously to applicants can reduce the number of unsynchronised finalisations. Units must therefore constantly discuss which applications to evaluate first. Having Units that are ahead of others in terms of evaluations would not result in registration; rather, additional personnel can be provided to the Units with the most work.
- With the synchronisation between Units executed, the finalisation of an application would be at similar times and properly monitored by the administrative Unit, now called the Health Product Authorisation (HPA) Unit.
- Inspections must be undertaken at the beginning of the process, and the status of the manufacturer must be established before scientific evaluations can be conducted.
- Increased frequency of registration meetings from six-weekly in the MCC process to weekly in the RBA process.

The last two solutions above were utilised in the BCP and RBA procedures, resulting in substantial improvements of the timeframes to 125 and 61 calendar days, respectively. RBA Phase 2 study saw a reduced timeframe of 33 days since most of the applications were already finalised by the other Units.

### Finalisation timeframe

Finalisation is the conclusion of an assessment by each respective Unit before registration. The finalisation timeline facilitates a comparison of the three processes utilised by SAHPRA between 2011 and 2022. The timeline was reported as 1470, 501, and 68 calendar days, for the MCC process, BCP process, and RBA phase 1 process, respectively, as depicted in Fig. 2. The median finalisation time of 73 calendar days was observed for the RBA phase 2 pilot study which consisted of a larger sample of 159 applications with a similar process as RBA phase 1. The finalisation time for the RBA process was drastically shortened, which is largely attributed to the strategic refinement, implementation of efficiencies, assessment style and ongoing monitoring of the registration process. The detailed examination of the MCC process enabled the authority to clearly identify the root causes inside the process; once these were discovered, the optimised and efficient RBA procedure was developed and piloted. The results clearly demonstrate that this procedure would reduce the backlog that has accumulated over time. It is crucial that each stage of the RBA process, as depicted in Table 1, has a precise deadline and monitoring mechanism to guarantee that these timelines are adhered to. The upscaling to 159 applications of the RBA procedure confirmed its repeatability and reproducibility with similar median timelines obtained. This robust procedure can therefore be utilised by other agencies who may have a backlog or want to optimise their registration process.

### Registration/approval timeframe

It was determined that the median approval/registration time between 2011 and 2017 was 2092 calendar days. Relative to other regulatory authorities, such as TGA with 244 calendar days for 85 applications in 2021 and ANVISA with 795 days between 2013 and 2016, the calculated median time for the MCC process was exceptionally long. [1, 9] This approval time was recorded as 591 calendar days for the BCP but was reduced to 511 calendar days for the RBA process. The median approval time for the RBA is due to the substantial amount of time the application waited in the queue for allocation. These applications had already been resubmitted early to mid-year 2020 and were awaiting allocation until September 2021. Therefore, almost 18 months had lapsed. This was deduced from the observed calculation of the median finalisation timeline of 68 days, thus, the remaining 443 days were attributed to applications waiting in line for allocation.

## Conclusion

This study identified the root causes which led to the formation of a backlog in the investigation of the MCC process. The factors were identified as inefficient processes employed, lack of monitoring and control, insufficient skilled staff for conducting the scientific assessments and limited review pathways employed. The most critical root cause was identified as the lack of monitoring and control by the authority in each step of the registration process which inevitably led to lengthy approval times. Comparison with the Brazilian authority also revealed that the claimed timeframes for the period 2011–2017 are much longer and must be substantially reduced to provide South African citizens with expedited access to medicine. The implementation of the BCP in 2019 introduced measures and resources that allowed for careful monitoring of the process. These contributed to reducing the reported end-to-end registration timelines, but they continued to remain longer than those reported by other authorities, and the targeted timelines were not met. In addition, the authority continued to develop a backlog despite the implementation of the process; consequently, more optimisation and refinement was required to meet the reduced timelines. The RBA approach was then piloted in 2021 and 2022, and its findings were much better than those of the previous two processes. A finalisation timeline of 68 and 73 calendar days was reported for RBA Phase 1 and 2 pilot studies, respectively, which is significantly shorter than the 1470 and 501 days indicated for the MCC and BCP processes. This rigorous RBA approach may also be used by regulatory agencies throughout the world to alleviate a backlog or to improve the efficiency of the existing process.

## Supplementary Information

**Additional file 1: Table S1.** The overview and approval times of the samples used in the Backlog clearance project and Risk-based assessment processes.

## Data Availability

Data not available due to privacy and confidentiality restrictions.
