# Supplementary material for: Regulatory registration timelines of generic medicines in South Africa: Assessment of the performance of SAHPRA between 2011 and 2022
Source: J Pharm Policy Pract. 2023 Mar 2;16:34. doi: 10.1186/s40545-023-00537-0 (PMC9983237; doi:10.1186/s40545-023-00537-0)
Supplement: Supplementary file 1 — Additional file 1: Table S1. The overview and approval times of the samples used in the Backlog clearance project and Risk-based assessment processes. [file 40545_2023_537_MOESM1_ESM.docx]

| Resubmission window | Resubmission window | Registration process | Sample size | Median finalisation time (calendar days) | Median approval time (calendar days) |
| --- | --- | --- | --- | --- | --- |
| RW1 | Human Immono deficiency Virus  Tuberculosis  Hepatitis | Backlog Clearance Project | 129 | 501 | 591 |
| RW5 | Maternal and newborn health  Diabetes  Malaria  Priority APIs |  |  |  |  |
| RW6 | Respiratory system diseases |  |  |  |  |
| RW8 | Haematological / immunological diseases  Analgesics & NSAIDs1 | Risk-Based Assessment Phase 1 | 63 | 68 | 110 |
| RW10 | Endocrine, nutritional and metabolic diseases  Digestive system diseases | Risk-Based Assessment Phase 2 | 159 | 73 | 95 |
| RW11 | Musculoskeletal system and connective tissue diseases  Skin and subcutaneous tissue diseases |  |  |  |  |
| RW12 | Eye and adnexa diseases  Ear and mastoid diseases  Other |  |  |  |  |

**Additional file 1**

**Table S1** The overview and approval times of the samples and used in the Backlog clearance project and Risk-based assessment processes
